# Pathways to professional mental care in the Swiss young adult community: a case–control study

**DOI:** 10.1007/s00406-024-01757-4

**Published:** 2024-03-01

**Authors:** N. Osman, C. Michel, B. G. Schimmelmann, L. Schilbach, E. Meisenzahl, F. Schultze-Lutter

**Affiliations:** 1 Clinic for Psychiatry and Psychotherapy/LVR-Clinic Duesseldorf, Medical Faculty and University Hospital Duesseldorf, Heinrich Heine University Duesseldorf, Duesseldorf, Germany; 2 University Hospital of Child and Adolescent Psychiatry and Psychotherapy, University of Bern, Bern, Switzerland; 3 University Hospital of Child and Adolescent Psychiatry, University Hospital Hamburg-Eppendorf, Hamburg, Germany; 4 Medical Faculty, Ludwig Maximilians Universität, Munich, Germany; 5 Department of Psychology, Faculty of Psychology, Airlangga University, Surabaya, Indonesia

**Keywords:** Active help-seeking behavior, Point-of-contact, Mental health problems/disorders, Community study

## Abstract

Treatment success for mental health (MH) problems depends, among others, on the timeliness of help-seeking. Therefore, we studied the effect of symptoms and reasons for help-seeking on the point-of-contact and the most intensive professional treatment in a community sample. Participants were recruited as part of the ‘Bern Epidemiological At-Risk’ (BEAR) study on 16–40-year-old community persons of the Swiss canton Bern. Of the 2,683 participants, 615 (22.9%) reported at least one instance of help-seeking for MH problems and were selected for the presented analyses. Help-seeking behavior was assessed by a modified version of the ‘WHO pathway-to-care questionnaire’, from which the outcome ‘most intensive MH professional contact’ was generated. The effect of symptoms and reasons for help-seeking were analyzed in separate models using path analyses. Most help-seeking persons sought MH professional help (*n* = 405; 65.9%) with a high number of medical pre-contacts (*n* = 233; 37.9%). The ‘most intensive MH professional contact’ was provided after an average of 1.47 contacts. Both models showed negative associations between non-MH professional pre-contacts and the most intensive, likely most adequate MH treatment. In the symptom model, ‘substance misuse’ and ‘central-vegetative problems’ increased the general likelihood of MH professional contact. Our findings highlight the importance of the first point-of-contact in pathways to adequate MH care and, when seeking help from non-MH professional, of quick referrals to MH professionals. Awareness campaigns or training of health professionals, such as general practitioners, may support timely contact with MH professionals to improve diagnosis, prognosis, and outcome.

**Supplementary Information:**

The online version contains supplementary material available at 10.1007/s00406-024-01757-4.

## Introduction

Persons with mental health (MH) problems often do not seek help at all or seek help in a delayed manner, which leads to more severe symptomatology, higher costs, and poorer outcome [1–4]. This results in a heavy burden to society, as approximately 30% of all persons worldwide develop a mental illness requiring MH professional treatment at some point in their lives [5].

Seeking formal help for MH problems most often takes place in outpatient settings, with general practitioners (GPs) frequently representing the first point-of-contact, thus serving as an important gatekeepers to MH services [6–8], especially for persons with lower education and/or income, and older persons [9]. In addition, informal help, such as family and friends or the internet, represent important points-of-contact for MH problems [10–12].

Mood, anxiety (excluding specific phobia), and psychotic disorders are one of the main reasons for help-seeking in general, while persons with substance misuse, eating disorders, and specific phobia are least likely to seek help [6, 8, 13–16]. In case of mood and anxiety disorders, the route to psychiatric help is often through primary care (i.e., GPs), while persons with psychotic disorders are more likely to seek MH professional help directly [9, 17]. Yet, a considerable number of patients remains in primary care [17], leading to longer delays in the delivery of appropriate guideline-based psychiatric-psychological treatment, and thus longer and increasingly severe symptomatology and poorer treatment outcomes [3, 18, 19].

Previous literature on the point-of-contact for MH problems and reasons for help-seeking focused on clinically relevant symptoms of specific mental disorders for which help was sought in patient samples [15, 17, 20] but—to the best of our knowledge—no study has used the most intensive MH treatment as a proxy measure for adequate treatment as outcome. To additionally map the personal reasons of help-seeking in a large community sample, we independently examined the effects of personal reasons for help-seeking and symptoms initiating help-seeking on the point-of-contact for MH problems, in particular on the most intensive MH professional treatment, thereby controlling for sex, age, and family history of mental disorders. In line with the literature, we expected that depressive and anxiety symptoms would be a main reason to seek help in primary care.

## Methods

### Sample and study design

The data of this study comes from the baseline assessment of the ‘Bern Epidemiological At-Risk’ (BEAR) study, a randomly selected representative population telephone study in the semi-rural canton Bern, Switzerland (for details see Online Resource sText1, sFigure1 and [21]). After randomly drawing potential 16- to 40-year-old participants from the population register of Bern, potential participants were first contacted by an information letter. Participants’ informed consent equaled participation in the telephone survey. Potential participants with past or present psychosis, and insufficient language skills in German, French, English, or Spanish were excluded. Altogether, 2683 participants (response rate: 63.4%) were interviewed between June 2011 and November 2014 about mental health problems and help-seeking. Of these, 615 (22.9%) participants reported at least one instance of help-seeking for mental health problems and represent the sample for this study. The BEAR study was carried out in accordance with the latest version of the Declaration of Helsinki and was approved by the local ethics committee of the University of Bern (No. 172/09).

### Assessments

Help-seeking behavior was assessed by a modified version of the ‘WHO pathway-to-care questionnaire’ [22–24] that has been widely used in previous studies of help-seeking behavior [25–27].

Based on this questionnaire, a MH professional contact was defined as help-seeking at psychotherapeutic/psychiatric practices or services, e.g., in school or at work, or psychiatric hospitals, emergency rooms, or outpatient units. For the present analyses, help-seeking pathways reported by participants were evaluated by help of the following variables:

- The ordinal outcome variable ‘most intensive MH professional contact’ was generated by coding the type of the most intensive MH professional contact from all help-seeking contacts as follows: 0 = ‘no MH professional contact’, 1 = ‘short outpatient contact (≤ 4 weeks)’, 2 = ‘long outpatient treatment (> 4 weeks, and approximately monthly or sporadically over several years)’, 3 = ‘longer inpatient treatment’. In the case of multiple MH professional contacts, the most intensive contact was selected based on institution and treatment duration, whereby long-term outpatient treatment was considered as more intensive than short-term inpatient treatment if its duration was at least three times longer.
- ‘Contact number’ describes which contact was the ‘most intensive MH professional contact’. If the same kind of MH professional contacts occurred in different pathways in succession, the number of the first contact of the series was chosen.
- Five dichotomous pre-contact variables, evaluating presence/absence, were created by evaluating the contacts prior to ‘most intensive MH professional contact’. In case of no report of professional contact, all non-professional help-seeking contacts were recorded in the corresponding variable, with rating of multiple categories possible.
- ‘No pre-contact’ if ‘most intensive MH professional contact’ was the first contact.
- ‘Other pre-contact’ if help was sought at religious organizations, legal services, schools, the police, or other non-medical/psychological, e.g., legal services.
- ‘Low-threshold pre-contact’ if help was sought at social services, educational counseling, telephone help-line, or other health-related services.
- ‘Medical pre-contact’ if help was sought from GPs, hospitals, emergency rooms, private clinics, or other non-MH medical practices.
- ‘MH professional pre-contact’ in case of less intense MH professional contacts before ‘most intensive MH professional contact’.
- The ‘duration of the contact’ in weeks describes how long the most intensive MH professional contact lasted. If the same kind of MH professional point-of-call was sought in several pathways in succession, the durations of the corresponding pathways were added up. If participants indicated ‘approximately monthly over several years’, 24 weeks were assumed and added. If participants indicated ‘sporadically over several years’, 10 weeks were assumed and added.
- ‘Latencies’ in pathway-to-care were assessed by the question “Was there any latency to pre-treatment or symptom onset (at first help-seeking)?” and binary coded for absence/presence.
- Main reason for help-seeking was assessed by five mutually exclusive categories coded for affirmation/negation: ‘worry, anxiety’, ‘advice from another person’, ‘impression of being ill/mentally disturbed and in need for help’, ‘unusual problems in daily life’, and ‘other reasons’.
- Symptoms causing help-seeking were assessed by the open question “What was the main problem for which you sought help?”. Responses were scored for presence in two categories: clinical high risk of psychosis (CHR) symptoms (i.e., attenuated and transient psychotic symptoms, and cognitive and perceptual basic symptoms included in CHR criteria [28] and non-specific symptoms/problems (see Table 2 for single symptoms), and other symptoms, not explicitly listed in the other two categories (e.g., signs of eating disorders or other psychosomatic complaints).

### Statistical analyses

Initially, an orthogonal explorative factor analysis (EFA) with varimax rotation based on a Pearson’s correlation matrix for the CHR and unspecific symptoms was computed to obtain independent factors. Sampling adequacy for each analysis was checked by the Kaiser–Meyer–Olkin measure [29] and Bartlett’s test of sphericity [30]. Reliability of the factors was computed using Cronbach’s alpha [31] and composite reliability [32]. The factors were included in further analyses as the sum score of their items.

Path models were computed using the diagonally weighted least squares estimator (DWLS) to estimate the model parameters. The weighted least squares mean and variance adjusted estimator (WLSMV) was used to estimate robust standard errors and a mean- and variance-adjusted test statistic [33]. The comparative fit index (CFI ≥ 0.95), the root mean square error of approximation (RMSEA ≤ 0.06), and the standardized root mean square residual (SRMR ≤ 0.08) were used to evaluate model fits [34, 35]. Usefulness of the χ^2^-statistic as a fit indicator is limited by its sensitivity to sample size and its tendency to reject models in large samples like ours [34]. Therefore, we followed the ‘2-index presentation strategy’ by Hu and Bentler [36] that suggests that a path model should be regarded as well fitting, if RMSEA and its 90% confidence intervals are ≤ 0.06, and SRMR ≤ 0.08.

Statistical analyses were conducted in R using package ‘lavaan’ for path models [37] and package ‘sempower’ for power analysis [38].

## Results

### Symptom factors

Ten of the thirty-four symptoms (nine of them CHR symptoms) were excluded because they were either not reported by any or just by one person (see Online Resources Table 1). The sampling adequacy with the remaining 24 symptoms was ‘middling’ (KMO = 0.726) according to Kaiser [29]. Bartlett’s test of sphericity (*χ*^*2*^(276) = 1724.36, *p* < 0.001) indicated that correlations between items were sufficiently large for the EFA [30]. The overall factor solution explained 25% of variance and both the Kaiser’s criterion and the scree plot (see Online Resources Fig. 2) converged on five factors. Orthogonal EFA with varimax rotation revealed the following five independent factors: ‘tension’, ‘depressiveness’, ‘social problems’, ‘substance misuse’, and ‘central-vegetative problems’ (Table 1). Six symptoms could not be clearly assigned to any factor because of low loadings on each factor (< 0.30; Table 1). Thus, the factors with scale reliabilities in a very good range were based on 18 symptoms (Table 1).

**Table 1:** Results of the explorative factor analysis of the 24 symptoms reported by participants for help-seeking (*N* = 615)

Items	Factor 1: tension	Factor 2: depressiveness	Factor 3: social problems	Factor 4: substance misuse	Factor 5: central-vegetative problems	Communality
Worries	**0.66**	0.14	0.07	– 0.03	0.09	0.47
Tension	**0.66**	0.29	0.14	0.00	– 0.02	0.55
Anxiousness	**0.46**	0.19	0.02	– 0.02	0.11	0.26
Headaches	**0.35**	0.26	0.04	0.09	– 0.25	0.26
Withdrawal behavior	0.03	**0.59**	0.04	0.05	– 0.04	0.35
Depressive mood	0.15	**0.52**	0.03	0.09	0.10	0.31
Self-confidence issues	0.21	**0.45**	0.00	0.03	0.25	0.31
Lack of energy	0.17	**0.43**	0.27	0.02	0.16	0.31
Hypersensitivity	0.10	0.08	**0.67**	– 0.03	– 0.04	0.47
Antisocial behavior	0.02	0.07	**0.50**	– 0.04	0.13	0.27
Irritability	0.16	0.21	**0.48**	0.02	0.10	0.31
Memory problems	0.06	0.06	**0.39**	– 0.01	– 0.06	0.16
Alcohol misuse	0.07	– 0.05	0.02	**0.96**	0.15	0.94
Substance misuse	– 0.06	– 0.01	– 0.03	**0.31**	0.00	0.10
Guilt feelings	0.11	0.20	– 0.08	0.09	**0.43**	0.25
Loss of libido	0.20	– 0.04	0.32	0.07	**0.38**	0.30
Appetite or sleep disturbances	0.21	0.25	0.03	0.00	**0.35**	0.23
Self-harm	0.01	0.03	0.06	0.17	**0.33**	0.14
*Cognitive basic symptoms (CHR)*	– 0.04	– 0.05	0.00	– 0.01	– 0.02	0.00
*Expansive mood/mania*	0.03	0.06	– 0.01	0.16	0.02	0.03
*Obsessive–compulsive symptoms*	– 0.05	– 0.02	0.09	– 0.01	– 0.01	0.01
*Other affective changes*	0.01	– 0.02	– 0.03	– 0.03	0.05	0.00
*Other behavioral abnormalities*	0.05	– 0.02	– 0.02	– 0.01	– 0.09	0.01
*Couple or family problems*	– 0.06	– 0.18	– 0.07	0.03	0.01	0.04
Eigenvalue	3.39	1.71	1.57	1.25	1.22	
Cronbach’s alpha	0.86	0.82	0.90	0.82	0.82	
Composite reliability	0.87	0.82	0.91	1.04	0.82	

Items given in Italics could not be assigned to any factor. Values given in bold indicate the affiliation of item to the factor

### Sample characteristics

Sociodemographic characteristics did not differ between participants with (*n* = 405) and without (*n* = 210) MH professional help-seeking behavior except for marital status (Table 2). Clinical characteristics differed in group frequencies of latencies within pathways-to-care, of low-threshold and medical pre-contacts, of seeking help by ‘advice from another person’ as well as of naming ‘tension’ and ‘central-vegetative problems’ as causes of help-seeking.

**Table 2:** Sociodemographic and clinical characteristics of the sample

	No MH professional help-seeking (*n* = 210; 34.1%)	MH professional help-seeking (*n* = 405; 65.9%)	Total sample (*N* = 615)	Statistics; effect size
Sex: male,* n* (%)	84 (40.0%)	142 (35.1%)	226 (36.7%)	*χ*^*2*^(1) = 1.246, *p* = 0.252; *V* = 0.049
Age, median (mean ± SD)	35 (32.56 ± 6.30)	34 (31.85 ± 6.99)	34 (32.09 ± 6.77)	*U* = 43,979, *p* = 0.486; *r* = − 0.001
Nationality: Swiss,* n* (%)	197 (93.8%)	380 (93.8%)	577 (93.8%)	*χ*^*2*^(1) < 0.001, *p* = 1; *V* < 0.001
Education^a^,* n* (%)				
ISCED 1	0 (0.0%)	1 (0.2%)	1 (0.2%)	
ISCED 2	4 (1.9%)	20 (4.9%)	24 (3.9%)	*χ*^*2*^(6) = 5.456, *p* = 0.482; *V* = 0.094
ISCED 3	13 (6.2%)	16 (4.0%)	29 (4.7%)	
ISCED 4	2 (1.0%)	5 (1.2%)	7 (1.1%)	
ISCED 5	120 (57.1%)	227 (56.0%)	347 (56.4%)	
ISCED 7	67 (31.9%)	127 (31.4%)	194 (31.5%)	
ISCED 8	4 (1.9%)	9 (2.2%)	13 (2.1%)	
Employment: yes,* n* (%)	205 (97.6%)	387 (95.6%)	592 (96.3%)	*χ*^*2*^(1) = 1.113, *p* = 0.264; *V* = 0.052
Marital status,* n* (%)				
Unmarried	94 (44.8%)	228 (56.3%)	322 (52.4%)	*χ*^*2*^(2) = 17.929, *p* < 0.001; *V* = 0.171
Married or registered partnership	108 (51.4%)	141 (34.8%)	249 (40.5%)	
Separated, divorced or widowed	8 (3.8%)	36 (8.9%)	44 (7.2%)	
Mental health disorder in family: yes,* n* (%)	113 (53.8%)	244 (60.2%)	357 (58.0%)	*χ*^*2*^(1) = 2.096, *p* = 0.143; *V* = 0.062
Mental health problems^c^,* n* (%)				
No mental health problem	83 (39.5%)	168 (41.5%)	251 (40.8%)	*χ*^*2*^(2) = 5.218, *p* = 0.074; *V* = 0.092
Only mental health problem, no mental health disorder^d^	89 (42.4%)	138 (34.1%)	227 (36.9%)	
Mental health disorder	38 (18.1%)	99 (24.4%)	137 (22.3%)	
Latency before any help-seeking contact: yes,* n* (%)	127 (60.5%)	192 (47.4%)	319 (51.9%)	*χ*^*2*^(1) = 8.945, *p* = 0.002; *V* = 0.124
Contact number^e^, median (mean ± SD)	0 (0.00 ± 0.00)	1 (1.45 ± 0.74)	1 (0.95 ± 0.91)	*U* = 0, *p* < 0.001; *r* = − 0.878
0	210 (100.0%)	0 (0.0%)	210	χ^2^(5) = 613, *p* < 0.001; V = 1
1	0 (0.0%)	267 (65.9%)	267	
2	0 (0.0%)	106 (26.2%)	106	
3	0 (0.0%)	18 (4.4%)	18	
4	0 (0.0%)	10 (2.5%)	10	
5	0 (0.0%)	2 (0.5%)	2	
Duration of contact (in weeks), median (mean ± SD)	0 (0.00 ± 0.00)	20 (35.62 ± 75.48)	6 (23.46 ± 63.51)	*U* = 0, *p* < 0.001; *r* = − 0.837
Pre-contact^f^: yes,* n* (%)				
No	0 (0.0%)	267 (65.9%)	267 (43.4%)	*χ*^*2*^(1) = 241.990, *p* < 0.001; *V* = 0.631
Low-threshold	48 (22.9%)	4 (1.0%)	52 (8.5%)	*χ*^*2*^(1) = 82.649, *p* < 0.001; *V* = 0.373
Medical	125 (59.5%)	108 (26.7%)	233 (37.9%)	*χ*^*2*^(1) = 62.055, *p* < 0.001; *V* = 0.321
MH professional	0 (0.0%)	32 (7.9%)	32 (5.2%)	*χ*^*2*^(1) = 15.938, *p* < 0.001; *V* = 0.169
Other	51 (24.3%)	13 (3.2%)	64 (10.4%)	*χ*^*2*^(1) = 63.644, *p* < 0.001; *V* = 0.327
Reason for help-seeking: yes,* n* (%)				
Concern, anxiety	64 (30.5%)	119 (29.4%)	183 (29.8%)	*χ*^*2*^(1) = 0.035, *p* = 0.781; *V* = 0.011
Advice from other person	52 (24.8%)	134 (33.1%)	186 (30.2%)	*χ*^*2*^(1) = 4.157, *p* = 0.034; *V* = 0.086
Feeling of being ill and in need for help	23 (11.0%)	53 (13.1%)	76 (12.4%)	*χ*^*2*^(1) = 0.401, *p* = 0.519; *V* = 0.031
Unusual problems in daily life	37 (17.6%)	48 (11.9%)	85 (13.8%)	*χ*^*2*^(1) = 3.393, *p* = 0.064; *V* = 0.079
Other	34 (16.2%)	51 (12.6%)	85 (13.8%)	*χ*^*2*^(1) = 1.216, *p* = 0.221; *V* = 0.049
Symptom factor^f^: median number (mean ± SD), and any 1 symptom affirmed,* n* (%)				
Tension	0 (0.40 ± 0.82)	0 (0.55 ± 0.95)	0 (0.50 ± 0.91)	*U* = 39,066, *p* = 0.037; *r* = − 0.072
	49 (23.3%)	127 (31.4%)	176 (28.6%)	*χ*^*2*^(1) = 3.975, *p* = 0.039; *V* = 0.084
Depressiveness	0 (0.54 ± 0.86)	0 (0.69 ± 0.98)	0 (0.64 ± 0.94)	*U* = 39,334, *p* = 0.081; *r* = − 0.056
	75 (35.7%)	170 (42.0%)	245 (39.8%)	*χ*^*2*^(1) = 2.008, *p* = 0.141; *V* = 0.061
Social problems	0 (0.06 ± 0.26)	0 (0.09 ± 0.42)	0 (0.08 ± 0.38)	*U* = 42,391, *p* = 0.875; *r* = 0.046
	12 (5.7%)	24 (5.9%)	36 (5.9%)	*χ*^*2*^(1) < 0.001, *p* = 1; *V* = 0.004
Substance misuse	0 (0.04 ± 0.22)	0 (0.05 ± 0.25)	0 (0.05 ± 0.24)	*U* = 42,156, *p* = 0.599; *r* = 0.010
	7 (3.3%)	17 (4.2%)	24 (3.9%)	*χ*^*2*^(1) = 0.093, *p* = 0.667; *V* = 0.021
Central-vegetative problems	0 (0.07 ± 0.29)	0 (0.19 ± 0.49)	0 (0.15 ± 0.43)	*U* = 38,643, *p* = 0.001; *r* = − 0.124
	13 (6.2%)	62 (15.3%)	75 (12.2%)	*χ*^*2*^(1) = 9.903, *p* = 0.001; *V* = 0.132

^**a**^International Standard Classification of Education 2011 (no participants with ISCED 6) (https://www.datenportal.bmbf.de/portal/en/G294.html)

^**b**^Social and Occupational Functioning Scale (SOFAS, 0–100, lower scores indicate lower psychosocial functioning)

^**c**^Excluding specific phobia

^**d**^Rated when a screening question of the M.I.N.I. was affirmed but the full criteria were not met

^**e**^Statistics calculated with *n* = 613 persons due to two outliers with contact number = 7

^**f**^Multiple answers possible

### Path models

**Fig. 1:**
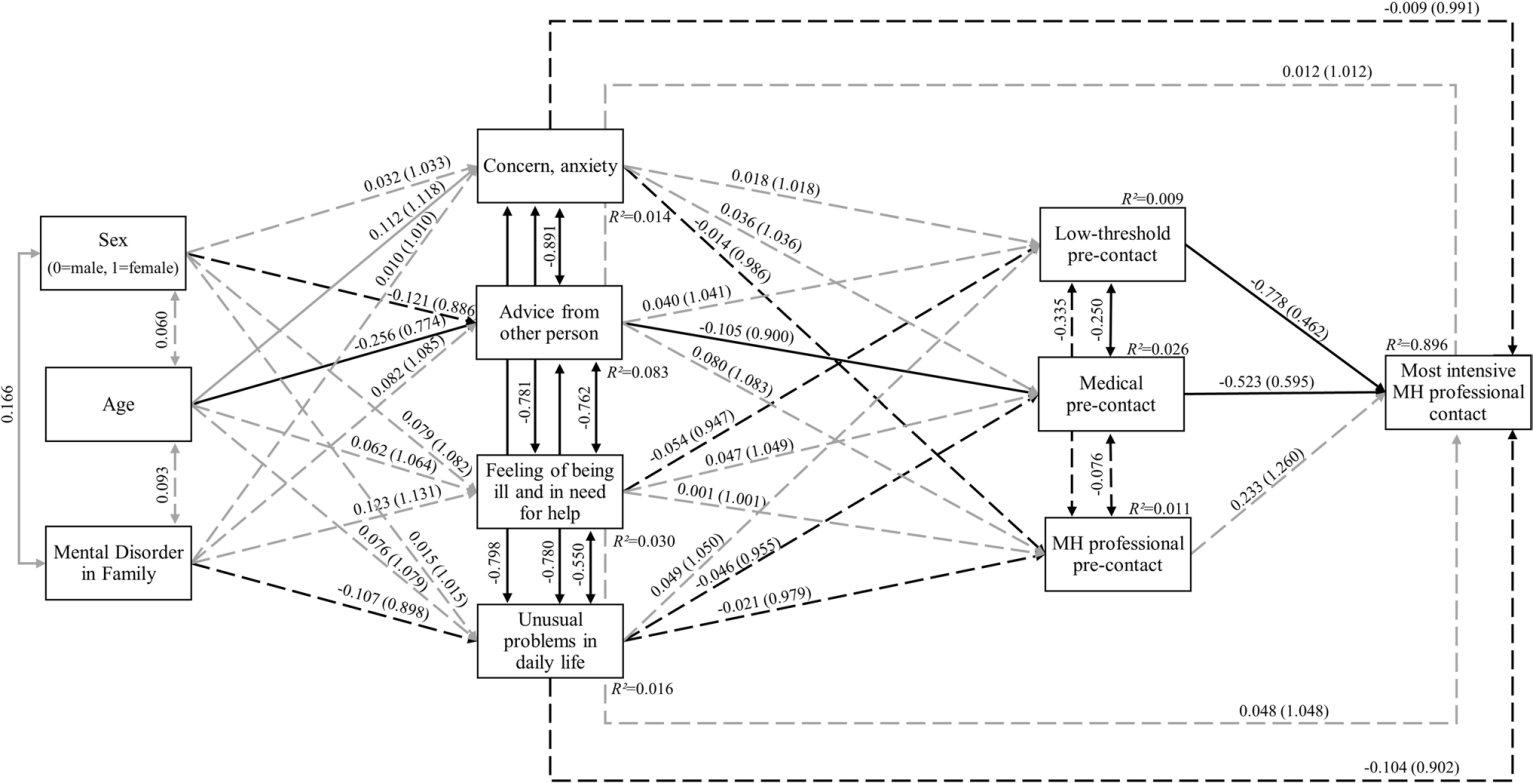
Path model including reasons for help-seeking (*N* = 615) with standardized path coefficients. Model fit indices: *χ*^*2*^(12) = 26.714 with *p* = 0.008, CFI = 0.954, SRMR = 0.054, RMSEA = 0.045 (90%CI = 0.022-0.068). Power = 0.956. Odds ratios in brackets. Solid lines indicate significant paths (*p* ≤ 0.05), dashed lines indicate non-significant paths (*p* > 0.05), grey indicates positive associations, black indicates negative associations

**Fig. 2:**
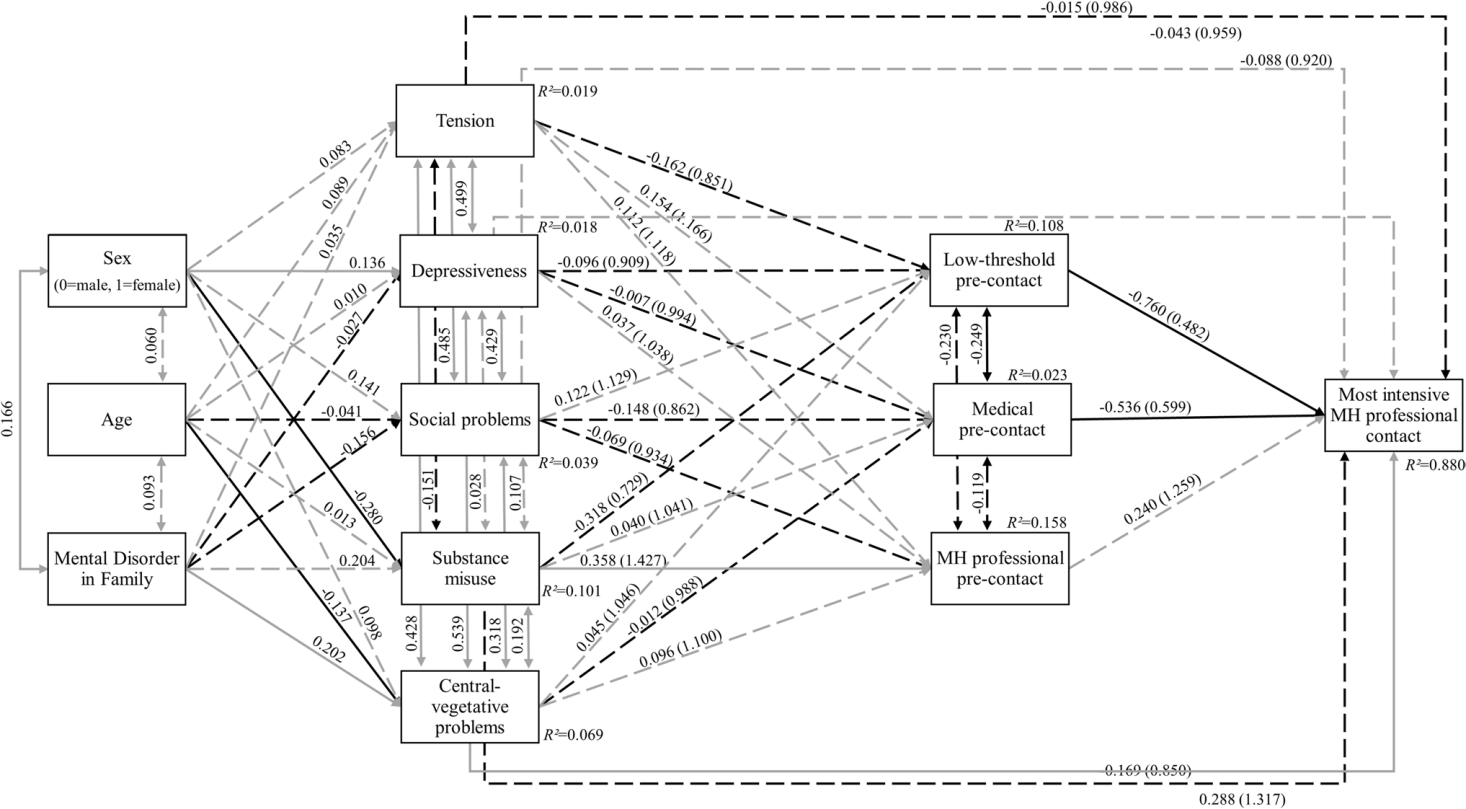
Path model including help-seeking initiating symptoms (*N* = 615) with standardized path coefficients. Model fit indices: *χ*^*2*^(12) = 22.920 with *p* = 0.028, CFI = 0.981, SRMR = 0.049, RMSEA = 0.038 (90%CI = 0.012–0.062). Power = 0.956. Odds ratios in brackets. Solid lines indicate significant paths (p ≤ 0.05), dashed lines indicate non-significant paths (*p* > 0.05), grey indicates positive associations, black indicates negative associations

Both path models, i.e., one with reasons for help-seeking (Fig. 1) and a second with symptom factors (Fig. 2) as predictors, showed excellent fit and power, explained high portions of each variance of the outcome ‘most intensive professional contact’ (*R*^*2*^ = 0.88 and *R*^*2*^ = 0.90). In both models, medical and low-threshold pre-contact were negatively associated with subsequent ‘most intensive professional contact’ and negatively with each other.

In the model including reasons for help-seeking (Fig. 1), ‘advice from other person’ was negatively associated with ‘medical pre-contact’. Further, younger participants more likely gave ‘advice from other person’, while older one more likely gave ‘concern, anxiety’ as the reason for help-seeking. All significant covariates between reasons for help-seeking were negative (Fig. 1).

In the model including help-seeking initiating symptoms (Fig. 2), ‘substance misuse’ and ‘central-vegetative problems’ were associated with ‘MH professional pre-contact’ and ‘most intensive MH professional contact’. ‘Central-vegetative problems’ were more likely named by younger participants and those with a family history of mental disorders, ‘depressiveness’ by women and ‘substance misuse’ by men. All significant covariances between symptom factors were positive (Fig. 2).

## Discussion

Mental disorders are often treated only with significant delay and/or inadequately. This contributes to their frequently poor outcome and high burden [3, 18, 19]. Thus, to understand and better promote pathways to adequate MH treatment, many barriers and facilitators, in particular MH literacy and attitudes, have been studied, frequently however with respect to help-seeking intentions rather than actual help-seeking behavior [39, 40]. Only few studies explored the type of MH problems initiating help-seeking and the reasons to seek help, and—to the best of our knowledge—no study has used the most intensive MH treatment as a proxy measure for adequate treatment as outcome. Thus, in this first-time path-analytic community study, we examined the effects of personal reasons for and of symptoms initiating help-seeking independent of the point-of-contact for MH problems and in particular on the most intensive MH professional treatment, thereby controlling for sex, age, and family history of mental disorders.

### The role of pre-contact at the highest intensity of MH treatment

Both models, i.e., the one including personal reasons and the one including symptoms, showed an equally excellent fit to the data. In our sample, the most intensive MH professional contact had a median duration of 20 weeks (*M* = 35.62, *SD* = 75.48) and was sought after an average of 1.47 contacts, indicating that the first MH professional contact is frequently also the one offering the most intensive treatment. The low numbers of MH professional pre-contacts (*n* = 32; 7.9%) likely explained the lack of significance of the positive association between MH professional pre-contact and the most intense MH professional treatment in both models. Although most help-seekers sought treatment from a MH professional point-of-contact (*n* = 405; 65.9%), in line with previous literature [6–9], medical help including GPs was the most frequent pre-contact (*n* = 233; 37.9%). Yet, in both models, the intensity of MH professional treatment was directly associated only or, in the symptom model, mainly with low-threshold and medical pre-contacts that lowered the intensity, i.e., the likelihood of receiving adequate guideline-based MH treatment, in each case. Because medical and low-threshold pre-contacts were also negatively associated with each other, this finding supports the reported tendency for help-seeking persons to often remain with already accessed point-of-contacts [9, 17]. This may be due to a sense of achievement or built trust with the respective point-of-contact [41]. As a result, help-seekers may be no longer motivated to seek or be referred to another point-of-contact or adequate guideline-based MH treatment [42].

The frequent medical pre-contacts, in particular with GPs, may be in part a result of the health care system in Switzerland. Switzerland has a mandatory health insurance (MHI), which is highly decentralized within the legal framework for managed competition in the statutory health system defined at the federal level [43]. The non-profit MHIs offer numerous types of MHI plans that differ with regard to the deductible and restrictions on their choice of health care providers. Patients generally can freely choose their physician but physician networks and health maintenance organizations (HMOs) increasingly contract with insurers to provide care, and the insurance premium of both the HMO and the GP plan is reduced by up to 20% [43, 44]. In 2012, about 20.8% of all insured persons in Switzerland were insured by either an HMO or GP plan [43], and in the canton Bern, where our study was conducted, the rate of HMO and GP plans was 23.5% in 2011, at the beginning of the data collection, steadily rising to 38.7% in 2021 [45, 46].

Outside these plans, ambulatory psychiatrists can be accessed directly by patients without GP referral, and care is reimbursed by MHI [43]. Yet, GPs generally play an important role and, in 2010, a good third of mental disorders in ambulant patients were diagnosed by GPs [43]. Until July 2022, psychotherapy by psychologists was reimbursed only if doctors (not necessarily MH specialists) with a specific license provided it themselves or delegated it to a psychologist who operates in the same practice as the doctor [43, 47]. Thus, persons in need or searching for psychotherapy reimbursed by their MHI would have been obliged to initially seek help from a medical doctor, especially a GP, which explains the high number of medical pre-contacts in our study to some degree.

In addition, despite the high ratio of psychiatrists per 100,000 persons of 0.42 in 2012 that was the highest ratio in Western countries, access to psychiatric services has remained difficult [43]. In 2010, a Swiss study compared psychiatrists and GPS with regard to the time delay involved in seeking medical attendance when psychiatric disorders begin to develop [48]. Actors simulating clinical symptoms of acute depressive or psychotic disorder are called psychiatrists or GPs asking for an appointment at the doctor’s earliest convenience due to their acute mental problems. Two thirds (68%) of the phone calls to the psychiatrists in private practice were answered by voice mail and personal contact was established with only 56% of the psychiatrists, compared to 21% answering by voice mail and 95% personal contacts with GPs. On average, 7.3 phone calls were necessary to successfully book an appointment with a psychiatrist, which was possible with only 30% of all the contacted psychiatrists [48]. Thus, making an appointment with a psychiatrist was much more difficult than making an appointment with a GP [48], this likely contributing additionally to the high number of medical pre-contacts in our study. The difficulties in getting in contact with a psychiatrist might lead to patients giving up on seeking help from MH professionals and turn to other doctors, mostly GPs, or other lower-threshold contacts.

These findings highlight the importance of the first point-of-contacts in pathways to adequate care of MH problems as well as of the health care system and/or individual MHI plan, which was not assessed in our study. They also highlight the necessity to improve quick referral to MH professionals, when seeking help from non-MH professionals. Awareness and information campaigns targeting both the general public and low-threshold and medical potential points-of-contact should support rapid access to MH professionals that should not be discouraged by frustratingly ineffective help-seeking attempts. Future studies will have to show, if the Swiss reform of the regulations for psychotherapy that allows psychological psychotherapists to work independently at the expense of MHI on a doctor’s order since July 2022 [47] improves faster contact with MH professionals.

### The role of reasons for help-seeking and their determinants in the choice of pre-contacts

All covariates between the four selectable reasons were negative and significant, which was likely caused by the fact that only one response could be selected. Younger age was related to more frequent help-seeking on advice from another person. These informal sources could be represented by parents, teachers, or friends [10–12] and likely reflect their concerns about the young person’s MH. Correspondingly, concerns and anxieties about own MH was related to older age in our sample. This is in line with reports that concerns about one’s health increase with age [49, 50], motivating help-seeking for both physical and MH problems.

When help was sought based on the advice of another person, the likelihood for a medical point-of-contact was reduced, while the likelihoods for MH professional or low-threshold help were increased—though not significantly.

### The role of help-seeking triggering symptoms and their determinants in the choice of pre-contacts

All covariates between symptoms were positive, which likely reflects the possibility to select multiple symptoms. Only the covariances of substance misuse with tension, depressiveness, and social problems did not become significant. Substance misuse was more likely a cause for help-seeking in men, while women were more likely to seek help for depressiveness. This is in line with reports of a higher prevalence of depression in women and of substance disorders in men [5, 51, 52]. Yet, while help for substance use problems was more likely sought from a MH professional, help-seeking for depressive symptoms was not specifically associated with any type of point-of-contact in our sample. Thus, our expectation that depressive and anxiety symptoms (the latter included in ‘tension’) would be especially associated with seeking help in primary care (included in ‘medical pre-contact’)—as reported from earlier studies [9, 17]—was not confirmed. This was despite the fact that symptoms of ‘depressiveness’ (*n* = 170, 42.0%) and ‘tension’ (*n* = 127, 31.4%) were most frequently stated as a reason for help-seeking in our sample, which is in line with reports of tension, anxiousness, and depressiveness being the symptoms for which help is generally sought most often [6, 8, 13–16].

Furthermore, younger age and a family history of mental disorder increased the likelihood of naming central-vegetative problems, including mostly appetite and sleep disturbances (*n* = 59, 65.6%) but also self-injury, as reasons for help-seeking. These kinds of problems are generally more common in younger persons [53, 54] and may be more observable to others, especially when they lead to severe physical consequences [55–57]. Thus, they may result in the observed significant association between ‘central-vegetative symptoms’ and ‘most intensive MH professional contact’. An additional exploratory path model of the four items of ‘central-vegetative problems’ on the most intensive MH professional contact indicated ‘appetite and sleep disturbances’ as the only significant predictor (*β* = 0.189, *p* < 0.001). Furthermore, the ‘true’ MH-related nature of central-vegetative problems may be better recognized in families with a history of mental disorders, explaining the positive association between ‘central-vegetative problems’ and a positive family history.

Both ‘substance misuse’ and ‘central-vegetative problems’ increased the general likelihood of MH professional contact, and, at descriptive level, 70.8% of persons with a substance use problem triggering help-seeking and 82.7% of persons with central-vegetative symptoms triggering help-seeking sought MH professional help. This link between substance misuse and MH professional service use is not in line with previous findings from Germany and Australia reporting lowest rates of lifetime MH service use for substance use disorders [8, 16]. However, as these studies had also included persons with no help-seeking behaviors, the focus of our analyses on persons with any kind of informal or formal help-seeking has likely biased our results toward high rates of help-seeking from MH services. Yet, contrary to ‘central-vegetative problems’, ‘substance misuse’ was only significantly linked to MH pre-contacts, indicating that the first MH professional contact has frequently not led to the most intensive treatment. This might reflect the reported gaps in continuity of care, such as limited initial treatment compliance [58], or limited access to treatment after completion of short-term inpatient medically managed withdrawal programs or acute emergency treatments [59, 60].

### Strengths and limitations

Our study has several strengths and limitations. Among the clear strengths are the investigation of active help-seeking behavior and the consideration of the most intensive, i.e., potentially guideline-compliant, treatment as the outcome (rather than only help-seeking intentions), and the use of complex path models enabled by a sufficiently large sample size of help-seekers of a representative sample. Nevertheless, more than 95% of the sample consisted of Swiss citizen between 16 and 40 years of age at baseline, so that the results can only be generalized to young and middle-aged adults in Western cultures, and similar universal private or public–private health insurance systems.

Another limitation might be related to the symptom factors. The overall low loadings of the EFA, many of which were below 0.50, may be due to the dichotomous nature of the variables. Also, the KMO values of 4 of 24 symptoms were below 0.50, which would have actually made them unsuitable for the EFA. Nevertheless, the analysis resulted in factors that were independent and meaningful in terms of content, with very good eigenvalues and reliabilities. Moreover, the factor ‘substance misuse’ consists of only two variables, which is below the recommended minimum number of four variables for a factor [61]. However, because of the construct immanence and meaningfulness of ‘substance misuse’ as a mental health disorder [5, 6], we accepted this factor.

### Conclusions and implications

Overall, our findings show that most persons seeking help for their mental health problems seek MH professional help. Yet, the negative associations between non-MH professional pre-contacts and the most intensive, likely most adequate MH treatment, and the low number of MH professional pre-contacts highlight the importance of the first point-of-contacts in pathways to adequate care of MH problems and, when seeking help from non-MH professional, of quick referral to MH professionals. Here, awareness campaigns or training of medical staff, such as general practitioners or pediatricians, can be used to encourage persons or, in case of minors, their parents to seek a MH professional point-of-contact as soon as possible to improve diagnosis, prognosis, and treatment outcome of their symptomatology.

## Supplementary Information

Below is the link to the electronic supplementary material.

Supplementary file1 (DOCX 925 KB)

## Data Availability

Data are available upon reasonable request from the senior author at frauke.schultze-lutter@lvr.de. Participants of the BEAR study gave informed consent for sharing of anonymized data.
