# Supplementary material for: Pathways to professional mental care in the Swiss young adult community: a case–control study
Source: Eur Arch Psychiatry Clin Neurosci. 2024 Mar 1;275(5):1509–20. doi: 10.1007/s00406-024-01757-4 (PMC12270957; doi:10.1007/s00406-024-01757-4)
Supplement: Supplementary file 1 — Supplementary file1 (DOCX 925 KB) [file 406_2024_1757_MOESM1_ESM.docx]

**Supplementary Material**

**to**

**Pathways to professional mental care in the Swiss young adult community: a case-control study**

**by**

**N. Osman, C. Michel, B. G. Schimmelmann, L. Schilbach, E. Meisenzahl and
F. Schultze-Lutter**

**Contents**

[**sText 1.** Study design, recruitment and representativeness of the sample of the
BEAR study [1]. 2](#_Toc136619184)

[**sFigure 1.** Survey outcome rates of the BEAR study at baseline according to the definitions of the American Association for Public Opinion Research [3]. 3](#_Toc136619186)

[**sTable 1.** Frequency and KMO values of all 34 symptoms (N=615). 4](#_Toc136619187)

[**sFigure 2.** Scree plot of the explorative factor analysis of the 24 symptoms. 5](#_Toc136619189)

[**References** 6](#_Toc136619190)

# **sText 1.** Study design, recruitment and representativeness of the sample of the BEAR study [1].

Study design

To increase response rates, contact was initially established using a one-page information letter. First telephone contact was attempted within two weeks of sending the letter. After detailed explanation of goals and proceedings of the study, participation in the telephone interview was considered as giving informed consent.

Inclusion criteria were being of eligible age (16-40 years) and being a main resident of Canton Bern (i.e., having a valid address and not being abroad during the assessment period). In addition, an available telephone number was required for eligibility. Participants were called up to 100 times over several months at various times and days, including Saturdays. Potential participants that were not reached within this time were considered as unknown eligible. Moreover, interviews were aborted prematurely when respondents had (i) a lifetime diagnosis of psychosis or (ii) insufficient language skills in German, French, or English.

The semi-structured interviews lasted 43 minutes on average (SD=20 minutes; range: 20–225 minutes). The BEAR study was carried out in accordance with the latest version of the Declaration of Helsinki.

Recruitment and response rates

From the 4471 eligible participants, 2857 interviews were conducted [1]. However, 125 (4.4%) of the 2857 interviews were aborted prematurely by the interviewer for insufficient language skills; 41 (1.4%) were aborted for a lifetime diagnosis of psychosis, which had not been diagnosed or treated in 19 cases [2]; and 8 (0.3%) were terminated prematurely by the participants themselves. Thus, according to the definitions of the American Association for Public Opinion Research [3], the contact rate was 94.8%, and the response rate, 63.4% with 2683 completed interviews [1]; see sFigure 1). Lack of time or interest was the main reason given by the 1350 (29.5%) refusers.

Representativeness of sample

The eligible sample was negligibly older than the 16- to 40-year-old general population of Bern (Cohen’s d=0.053), mainly because a non-significant higher number of available telephone numbers (landlines) was found for 36- to 40-year-olds [1]. Similar to the observation for the eligibility sample, the 2683 interviewees differed negligibly, i.e., at less than small effect size (Cohen’s d=0.040), from the 16- to 40-year-old general population of Bern in age distribution, but not in sex, nationality, and marital status. Consequently, as no response bias was detectable beyond the extremely small age-related inclusion bias, the participants were regarded as well representative of their age group [1].

# **

**

# **sFigure 1.** Survey outcome rates of the BEAR study at baseline according to the definitions of the American Association for Public Opinion Research [3].

# **sTable 1.** Frequency and KMO values of all 34 symptoms (N=615).

| **Symptom** | **Present (%)** | **KMO*** |
| --- | --- | --- |
| Depressive mood | 200 (32.52%) | 0.788 |
| Couple or family problems | 131 (21.30%) | 0.722 |
| Anxiousness | 105 (17.07%) | 0.786 |
| Worries | 97 (15.77%) | 0.760 |
| Tension | 87 (14.15%) | 0.805 |
| Lack of energy | 77 (12.52%) | 0.735 |
| Self-confidence issues | 60 (9.76%) | 0.812 |
| Appetite or sleep disturbances | 59 (9.59%) | 0.705 |
| Withdrawal behavior | 55 (8.94%) | 0.778 |
| Irritability | 24 (3.90%) | 0.758 |
| Headaches | 19 (3.09%) | 0.722 |
| Substance misuse | 19 (3.09%) | 0.516 |
| Guilt feelings | 17 (2.76%) | 0.662 |
| Hypersensitivity | 16 (2.6%) | 0.715 |
| Other affective changes | 12 (1.95%) | 0.432 |
| Alcohol misuse | 9 (1.46%) | 0.567 |
| Self-harm | 9 (1.46%) | 0.613 |
| Other behavioral abnormalities | 9 (1.46%) | 0.456 |
| Memory problems | 6 (0.98%) | 0.734 |
| Cognitive basic symptoms (CHR) | 5 (0.81%) | 0.636 |
| Loss of libido | 5 (0.81%) | 0.717 |
| Antisocial behavior | 5 (0.81%) | 0.648 |
| Expansive mood/mania | 4 (0.65%) | 0.461 |
| Obsessive-compulsive symptoms | 3 (0.49%) | 0.378 |
| Unusual perceptual experiences (CHR) | 1 (0.16%) |  |
| Hallucinations (CHR) | 1 (0.16%) |  |
| Perceptual basic symptoms (CHR) | 0 (0%) |  |
| Ideas of reference (CHR) | 0 (0%) |  |
| Unusual ideas and magical thinking (CHR) | 0 (0%) |  |
| Paranoid ideas (CHR) | 0 (0%) |  |
| Delusions (CHR) | 0 (0%) |  |
| Odd thinking and speech (CHR) | 0 (0%) |  |
| Formal thought disorder (CHR) | 0 (0%) |  |
| Odd behavior or appearance | 0 (0%) |  |

* Only calculated for symptoms with n>1.

#
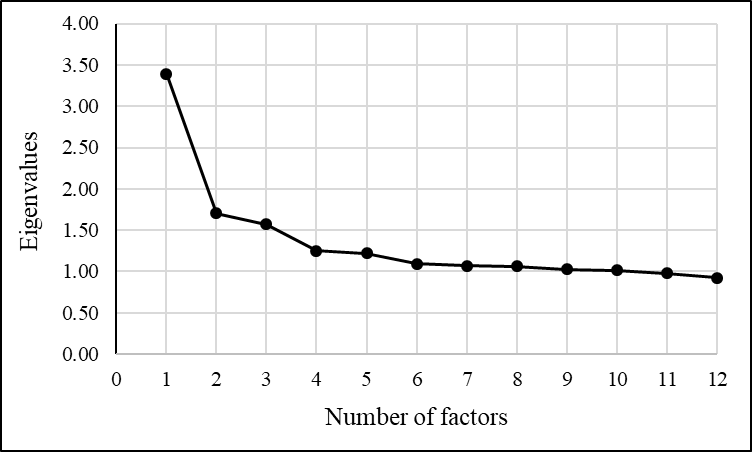


# **sFigure 2.** Scree plot of the explorative factor analysis of the 24 symptoms.

**References**

1. Schultze-Lutter F, Michel C, Ruhrmann S, Schimmelmann BG (2018) Prevalence and clinical relevance of interview-assessed psychosis-risk symptoms in the young adult community. Psychol Med 48:1167–1178. https://doi.org/10.1017/S0033291717002586

2. Michel C, Ruhrmann S, Schimmelmann BG et al (2018) Course of clinical high-risk states for psychosis beyond conversion. Eur Arch Psychiatry Clin Neurosci 268:39–48. https://doi.org/10.1007/s00406-016-0764-8

3. American Association for Public Opinion Research (2016) Standard Definitions: Final Dispositions of Case Codes and Outcome Rates for Surveys, 9th edn. AAPOR, Alexandria
